# The Role of Biopolymer-Based Materials in Obstetrics and Gynecology Applications: A Review

**DOI:** 10.3390/polym13040633

**Published:** 2021-02-20

**Authors:** Fauziah Jummaat, Esam Bashir Yahya, Abdul Khalil H.P.S., A. S. Adnan, Amaal Mohammed Alqadhi, C. K. Abdullah, Atty Sofea A.K., N. G. Olaiya, Munifah Abdat

**Affiliations:** 1 Management & Science University Medical Centre, University Drive, Off Persiaran Olahraga, Section 13, Shah Alam 40100, Malaysia; 2 School of Industrial Technology, Universiti Sains Malaysia, Penang 11800, Malaysia; essam912013@gmail.com (E.B.Y.); ck_abdullah@usm.my (C.K.A.); ngolaiya@futa.edu.ng (N.G.O.); 3 Faculty of Medicine, El Mergab University, Al Khums 00218, Libya; essam@asmarya.edu.ly; 4 Hospital Seberang Jaya, Jalan Tun Hussein Onn, Seberang Jaya, Permatang Pauh 13700, Malaysia; attysofea.8868@gmail.com; 5 Department of Preventive and Public Health Dentistry, Faculty of Dentistry, Universitas Syiah Kuala, Banda Aceh 23111, Indonesia; munifahabdat_dr@unsyiah.ac.id

**Keywords:** biopolymers, materials, obstetrics, gynecology, biomedical applications

## Abstract

Biopolymers have gained tremendous attention in many daily life applications, including medical applications, in the past few years. Obstetrics and gynecology are two fields dealing with sensitive parts of the woman’s body and her newborn baby, which are normally associated with many issues such as toxicity, infections, and even gene alterations. Medical professions that use screening, examination, pre, and post-operation materials should benefit from a better understanding of each type of material’s characteristics, health, and even environmental effects. The underlying principles of biopolymer-based materials for different obstetric and gynecologic applications may discover various advantages and benefits of using such materials. This review presents the health impact of conventional polymer-based materials on pregnant women’s health and highlights the potential use of biopolymers as a safer option. The recent works on utilizing different biopolymer-based materials in obstetric and gynecologic are presented in this review, which includes suture materials in obstetric and gynecologic surgeries, cosmetic and personal care products, vaginal health, and drug delivery; as well as a wound dressing and healing materials. This review highlights the main issues and challenges of biopolymers in obstetric and gynecologic applications.

## 1. Introduction

In a period of rapid growth of materials and medical knowledge and technological advancements, progressively more is expected to be learned regarding developing new materials to improve patients’ quality of life [1]. Obstetrics and gynecology are two fields dealing with sensitive parts of women’s bodies and their newborn babies [2]. Weakening the immune system upon pregnancy makes pregnant women more sensitive to the effect of different potentially toxic materials, including conventional polymers [3]. Many conventional synthetic or petroleum-based polymer-based materials are widely used in pregnant women’s daily lives, confirmed by many epidemiological and human monitoring studies their ability to cause serious health issues, including cancers [4,5,6]. The toxic effect of these materials is restricted to pregnant women, but it has been confirmed that such materials can induce genetic alterations, which may lead to a significant genetic deformity in future generations [7,8].

Biopolymers have been proposed to be a safer alternative to conventional polymers in many biomedical applications such as tissue engineering scaffolds [9,10], drug delivery [11], biosensing [12], wound healing [13], obstetrics, and gynecology [14,15]. The invention pertains to developing new innovational biopolymer-based materials still challenging. Many scientists joined the race to use more effective, less toxic, low cost, and sustainable materials for different medical applications [16]. Despite their safety, non-cytotoxic, and non-genotoxicity, biopolymers-based materials can also be efficiently be utilized after they serve the purpose they made for, without polluting the environment and cause significant health hazards as synthetic polymers based materials do [17]. Numerous studies have been published and reviewed in the past few years [18,19], regarding biopolymer-based materials [20], their properties [21], and applications [22], the medical applications of different biopolymers have also been extensively reviewed in many publications [23,24]. There is no review covering the role of biopolymer-based materials in obstetrics and gynecology application compared to conventional polymers. This review presents the health impact of conventional polymers on pregnant women upon exposure to cosmetics, personal care materials, therapeutics, etc. It proposes biopolymers as a safer alternative for different obstetrics gynecology applications that directly contact pregnant women and their pre- and post-delivery; highlighting the issues and challenges of utilizing biopolymer-based materials in cosmetic and pregnancy care, gynecological surgical sutures, vaginal care, and wound management materials.

## 2. Health Impact of Conventional Polymer-Based Materials in Obstetrics and Gynecology

Nowadays, synthetic polymers have become part of most of the materials in our lives, including food and beverages, clothes, baby-toys, daily used instruments, and even biomedical applications such as drug delivery systems and surgical equipment, and cosmetics personal care materials [25,26]. Some studies have linked these materials with potential health problems, especially to pregnant women and newborn infants [27,28]. Hormonally active agents or endocrine disruptors are a group of polymeric chemicals that have been related to critical health issues such as congenital disabilities, cancerous tumors, and other developmental disorders [29]. Pregnant women are the most affected group that have recently sound an alarm about endocrine disruptors. Noticing a startling trend in health issues in newborn babies, such as male babies with a congenital deformity of having urethra opening on the side instead of the tip of their penis [30]. The use of synthetic or non-biodegradable polymers generally represents better control in physicochemical and mechanical properties, but not safety [31,32]. The daily used materials, including cosmetics and personal care materials such as deodorants, lesions, creams, vaginal mucoadhesive, and other medical and surgical materials containing conventional polymers have been reported because of inflammation, cancers, fetus abnormalities, and genes alterations [33,34,35].

Biocompatibility has increasingly become a vital factor, especially in tissue-contacting applications. Various conventional polymers have been reported to be cytotoxic and cause low cellular viability upon direct contact with the conventional polymers [36,37]. Adhesive resin cements are increasingly used in modern dentistry. Nevertheless, released substances from resin materials have been shown to cause toxic cellular effects. Diemer et al. [38] investigated the effect of resin-cements against different cell-lines and reported that all their tested resin cements significantly reduced cell viability of human cells especially, osteoblastic cells demonstrated a tremendous increase of cytotoxicity after cement exposure. The authors suggested that the wide use of resin cement in every clinical situation should be scrutinized. In a different study, Çobanoğlu et al. [39] evaluated the cytotoxicity of polyethene and revealed a decrease in the cell proliferation index upon the cellular exposure to the polymer cytotoxic and genotoxic potential in human peripheral blood lymphocytes. The authors reported that polyethylene exposure caused chromosome instability in human lymphocytes.

### 2.1. Cosmetics and Personal Care Materials

Modern cosmetics often contain various polymeric and nano-sized components, which can penetrate human skin cells and cause many diseases [40]. During pregnancy, the body becomes weaker, in terms of anatomically, physiologically, and even immunologically, leading to an increase in foreign materials’ chance to cause adverse health effects [41]. Some studies revealed very high concentrations of polymeric and metallic debris particles inside the tissue surrounding the hip and knee replacements [42,43]. The presence of a high concentration of polymers inside the tissue confirms their penetration and restriction inside the cells, leading to inflammation, ultimately, osteolysis, and cancers [44,45]. Cationic polymers such as distearyldimonium chloride and benzyl dimethyl ammonium chloride are highly popular ingredients in hair products, which tend to be very substantive to the hair and difficult to remove [46]. Some epidemiological studies have detected a significant risk of conventional polymeric ingredients used nowadays. [47,48]. Fruijtier-Pölloth et al. [49] conducted a safety assessment for polyethene glycols and their commonly used derivatives in cosmetic products and revealed acute toxicity for such compounds, including skin irritation and sensitization, eye and mucosa irritation, carcinogenicity, reproductive toxicity, and even genotoxicity. In a different study, Biondi et al. [7] investigated the potential induction of chromosome aberrations by tetraethylene glycol in Chinese hamster epithelial cells. The authors reported a significant increase in the exchange rate between the sister chromatid in addition to chromosome damage. These chemicals’ ability to cause genetic changes upon their penetration inside the cells could turn the cells to either become carcinogenic or transfer the faulty genes to future generations [50,51]. Exposure to bisphenol A, which can be absorbed through the skin, has been significantly linked to neurological and reproductive damage [52]. Sugeng et al. [53] identified predictors of phthalate chemical levels in pregnant women in Australia. They revealed that higher phthalate levels in pregnant women were significantly associated with consuming tinned food, such as tomatoes and fish.

**Figure 1:**
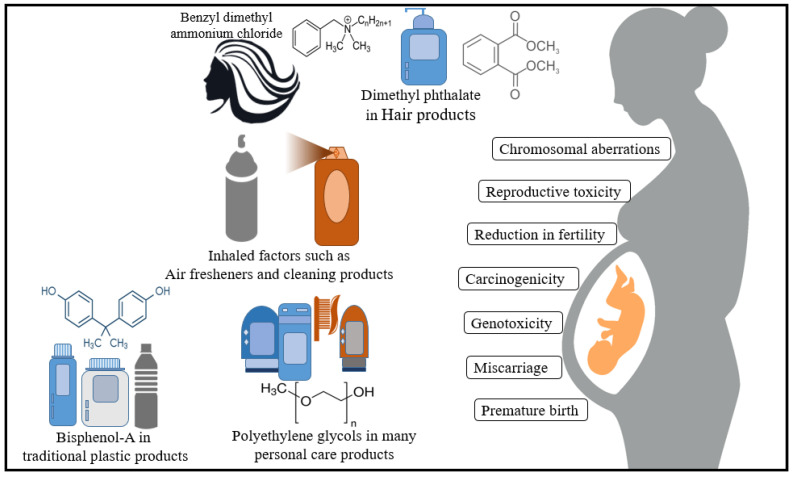
Illustration of the health risk of conventional polymers in cosmetic and personal care materials on pregnant women.

In contrast, the level of diethyl phthalate was considerably higher in women who use an air freshener. It has been reported that the exposure of using such synthetic materials affects offspring health and may lead to cancer, diabetes, obesity, and neurodevelopmental problems [54,55]. Conventional plastics and plastic-based materials that contain Bisphenol-A, which proved to cause a reduction in fertility, increase the chances of miscarriage in pregnant women, or even premature birth [56,57]. Refer to Figure 1 to summarize the effect of conventional polymers in cosmetic and personal care products on pregnant women.

### 2.2. Therapeutic Pharmaceuticals 

**Figure 2:**
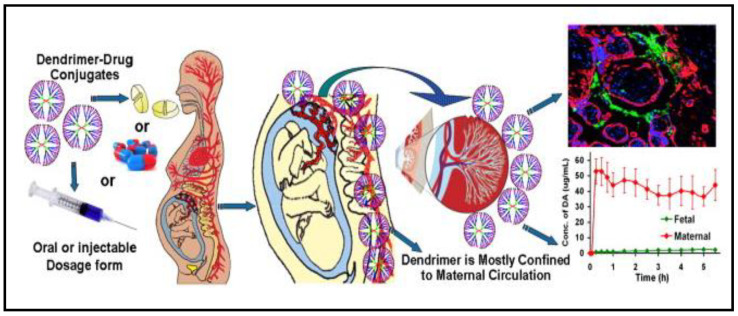
Schematic illustration of polyamidoamine dendrimers for drug delivery during pregnancy. Adapted from Menjoge et al. [63].

Poly(ethyl acrylate-co-methyl methacrylate-co-trimethyl ammonia-ethyl methacrylate chloride) or Eudragit RS 100 is a highly used synthetic polymer in gynecological drug delivery [58]. It has been used to deliver numerous vaginal antifungal, antiviral, antibacterial drugs, and other vaginal diseases in the form of nanocapsules and nanospheres [59]. Some studies have shown that Eudragit RS 100 may degrade into smaller fragments and remain in the body for a long time, leading to safety concerns as it accumulates inside the body and potentially induces immune disturbance [60,61,62]. Polyamidoamine dendrimers have been widely used in different drug delivery and biotechnology applications. Menjoge et al. [63] successfully developed polyamidoamine dendrimers drug carrier to be used during pregnancy as a novel approach for selectively delivering different therapeutics without significant transfer from pregnant women’s circulation to the fetus (Figure 2). Many studies revealed dendrimers’ possibility of undergoing endocytosis and crossing the cell membrane to reach intracellular localization [64,65]. Functionalization of polyamidoamine drug delivery system dramatically affected their ability to diffuse and penetrate the central nervous system tissues [66]. The dendrimer also was reported to induce dramatic apoptotic action and in vitro cell death of neurons [66]. This report suggests a potential health issue in large concentrations and for the long-term and the next generation, as these types of polymers can penetrate and remain inside the cells for a long period. 

Polyethene glycol is a petroleum-based polyether compound, has been used in various medical applications, including gynecological drug delivery, due to its lubrication ability and excellent moisture retention [67]. Although polyethene glycol is considered safe and almost non-toxic, researchers have recently noticed unsafe issues regarding this polymer. Polyethene glycol might has been reported to cause chronic oral toxicity in rats and humans, suggesting significant safety problems of these ‘safe’ materials [49]. In a different study, Liu et al. [68] evaluated the cytotoxicity of polyethene glycol derivatives on human cervical cancer cells. They revealed a potential hazard that shows that trimethylene glycol tends to be more toxic at high concentrations. Phthalates are a family of polymeric chemical compounds present in various pharmaceutical drugs and are thought to be hormonally active agents, which could cause endocrine disruptions [69]. Broe et al. [70] investigated the effect of phthalate exposure from different pharmaceutical drugs. They revealed that pregnant women who have been exposed to some phthalate polymer-containing drugs during the third trimester were highly associated with preterm birth. Other studies suggested that even environmental exposure of pregnant women to hormonally active agents such as phthalates polymer may increase preterm birth risk [71,72].

### 2.3. Surgical Sutures

Surgical sutures made from conventional polymers have been reported to induce a certain degree of an undesirable inflammatory reaction upon using them in obstetric and gynecologic surgeries, which is different based on the used material [73,74]. Lee et al. [75] evaluated the suture complication rates in addition to surgical outcomes of surgery (vaginal uterosacral ligament suspension) using synthetic polymers included monofilament polypropylene and multifilament polyester as sutures. The authors reported significant complications regarding using these synthetic polymers including suture erosion at the vaginal apex and granulation tissue, higher in multifilament polyester sutures [76]. Cytotoxicity of conventional polymers upon the direct contact of open wounds is the main cause of increasing inflammation reactions [74]. Apart from their ability to penetrate inside the cells, conventional polymers have been reported to induce the production of numerous cytokines, which may lead to disturbance in a particular tissue environment and cause undesirable effects [77]. Polyglycolic acid was introduced as a synthetic surgical suture in the early 1979s, followed by poly (lactic-co-glycolic acid) sutures [78]. Ceonzo et al. [79] investigated and revealed significant induction of local inflammatory response using polyglycolic acid-based sutures. Polyvinylidene fluoride is another synthetic polymer representing an attractive alternative to polypropylene for surgical sutures and a monofilament vascular suture [80]. Other synthetic polymers-based sutures have been used—including polypropylene, polytrimethylene carbonate, polydioxanone, etc.—they reported poor knot security [81,82]. The high stability and non-biodegradability of synthetic polymeric sutures make another operation’s need to remove the tissue’s suture. However, obstetric and gynecologic surgeries may not resist such a requirement, especially in diabetic or week immune women [83], giving the need for better options to avoid any potential complications.

### 2.4. Other Applications

Vitrification process requires cryoprotectant solutions to prevent ice crystals and increase the solution’s viscosity at low temperatures [84]. Different polymeric cryoprotectants have been used, including glycerol, ethylene glycol, propylene glycol, 1,2-propanediol, dimethylsulfoxide, sucrose, etc. [85]. Most of these cryoprotectants proved to have some toxicity and could cause some changes in the preserved cells of tissues, vitrifying on cooling at a smoothly repeatable rate. Faustino et al. [86] reported that ovarian tissue fragments could be cryopreserved to preserve females’ fertility by protecting their ovaries’ functions. Bari et al. [87] studied poly-vinyl pyrrolidone’s effect in vitrification solutions on vitrification of Buffalo oocytes. They revealed that poly-vinyl increases pyrrolidone concentration in the solution the number of cells reduced, in addition to causing morphological changes in the oocytes after vitrification. In a different study, Vizcarra et al. [28] reported that using synthetic polymers improves the quality and performance of vitrified ovarian tissue without testing their genetic or cytotoxic effect. However, Amorim et al. [88] evaluated the effect of using different vitrification solutions on human preantral follicles’ morphology. They concluded that vitrification solutions containing less toxic materials showed fast-penetrating and did not affect follicular morphology. In a recent study, Kokotsaki et al. [89] evaluated the impact of vitrification on human granulosa cell survival and its effect on gene expression. The authors used two different vitrification solutions; the first one contains DMSO. The second one contains polyethene glycol and revealed many dead cells and noticeable gene change variation. Santos et al. [90] summarized a plethora of DMSO cellular effects like reactive oxygen species scavenging, modulation of the cell cycle, apoptosis, and protein expression. Liu et al. [68] evaluated PEG-based monomers’ cytotoxicity and revealed obvious cytotoxicity only at high concentrations compared to low concentrations, which did not show any significant cytotoxicity. In many countries, the first choice for dental filling materials is tooth-colored polymers, which have been related to some concerns about their safety, especially in pregnant women [91]. The presence of endocrine disrupters in such fallings could pass through the placental barrier in the fresh filling process, putting the vulnerable fetus at risk [92]. Results from animal studies have indicated that bisphenol A has reproductive and developmental effects, in addition to systemic toxicity [93,94]. In a different study, Pfeifer et al. [95] reported the health effect of polymer-based direct filling materials on pregnant women and revealed that these polymers able to induce dental disturbance even after long period. Non-degradability of these polymers make them remain and possibly fracture to nano sized pieces and penetrate inside the cells, leading to serious health issues.

## 3. Biopolymer-Based Materials in Obstetrics and Gynecological Applications

**Figure 3:**
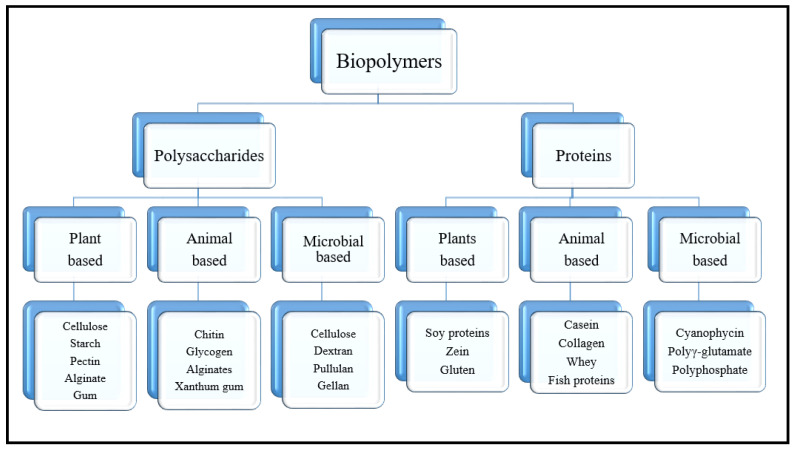
Classification of biopolymer based on their sources.

Biopolymers are naturally occurring polymers produced by living cells of animals, plants, and microorganisms, either polysaccharides, protein, or even polyesters (Figure 3) [96,97,98]. The ideal biopolymer for any medical application would have many characteristics, such as: non-toxic, does not evoke an inflammatory or immunological response, is easily sterilized, has an acceptable shelf life, and can be easily processed to its final form [26,99]. Many biopolymers have been proved for their non-toxicity, biocompatibility, and enhance cellular viability and proliferation. Ramphul et al. [100] reported that due to several OH groups’ presence, in many biopolymers such as cellulose, they possess high hydrophilicity and promote cellular interactions. Vartiainen et al. [101] investigated the cytotoxicity and biocompatibility of biopolymers-based tissue scaffolds. They revealed no cytotoxic effect on human or mouse cell lines scaffold did not cause any effects to the cells.

The application of biopolymer-based materials in obstetrics and gynecology have been a thrust area of research in the past few years due to the unique and superior properties that many biopolymers exhibit [102]. The current decade witnessed an increased use of biopolymeric based materials in the form of hydrogels, aerogels, films, sutures, surgical implants, examination materials, scaffolds for tissue engineering, and drug delivery, which can be attributed to the extraordinary and exceptional versatility that many biopolymers possess when compared to conventional petroleum, metal, or ceramic-based materials [103]. The successful utilization of biopolymers in many obstetrics and gynecological applications, including vaginal drug delivery [104], cosmetic and personal care products [105], examination equipment is an instrument as bioplastics [106], smart gynecological sutures, and wound healing products [107,108], etc., attracted researchers for more development to avoid using the conventional and health-hazardous polymers. 

### 3.1. Biopolymer-Based Materials in Cosmetics and Personal Care

**Figure 4:**
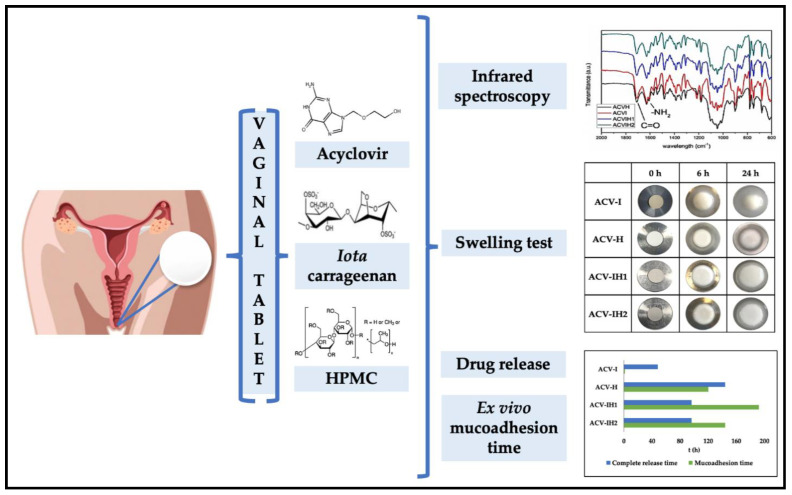
Fabrication of biopolymer-based vaginal tablet for antiviral delivery against sexually transmitted diseases. Adapted from Pacheco-Quito et al. [116].

Numerous biopolymer-based cosmetic ingredients are commercially available—including cellulose, starch collagen, keratin, and elastin—which have been applied in various cosmetic and personal care formulations [109]. Biopolymers play a critical role as thickening and moisturizer agents. A range of biopolymeric hydrogel has been fabricated for skin and hair care products [110]. Biopolymer based emulsions spread have been proved to be significantly better on the sensitive skin compared to those containing conventional based polymers [111]. They provide stringiness upon pick-up and comfort stickiness feeling to the skin [112]. Shakeri-Zadeh et al. [113] synthesized biopolymeric composites using chitosan and silver nanoparticles as antibacterial agents. The authors modified the conventional cotton tampon with their composite and revealed significant enhancement in absorption capacity and strong antibacterial activity. No erythema or edema was observed for modified tampon on the skin, indicating no sign of any dermal toxicity, suggesting great potential for upgrading the quality of regular feminine cotton tampon [113]. Genital herpes is globally common, especially in women of developing countries, affecting nearly 400 million people worldwide [114]. Pacheco-Quito et al. [115] fabricated vaginal tablets using a natural combination of the biopolymer hydroxypropyl methylcellulose and iota-carrageenan for delivery control the release of the antiviral drug acyclovir. The authors revealed the ability of tablets in controlling the release of acyclovir, which showed high mucoadhesive capacity through vaginal walls allowing the formulation to remain within the vaginal area long enough, leading to complete release of the antiviral drug (Figure 4). 

Collagen-based biocomposite was prepared from collagen/gelatin/hydroxyethyl cellulose as a natural formulation for skincare applications [116]. The biocomposite possessed higher swelling properties than commercial synthetic ingredients; the authors revealed this composite’s potentials in different cosmetic and dermatological applications as safer options for women with sensitive skin and during pregnancy. Chitosan is another biopolymer characterized by antimicrobial nature; it has been used in various skin and hair products, such as soaps, shampoos, permanent wave agents, rinses, and styling lotions, hair colorants, and hair sprays [117]. Polymeric polysaccharides and natural proteins have been utilized for various preparations for potential cosmetic uses [118]. Silk fibroin and keratin are two commonly used biopolymeric proteins that showed great potentials in cosmetic products. Zhu et al. [119] fabricated silk fibroin-based hydrogels with excellent mechanical properties for potential use in many biomedical and cosmetic applications. Refer to Table 1 to summarize the most used biopolymer-based formulations in cosmetic and personal care applications.

### 3.2. Biopolymer-Based Materials in Obstetrics and Gynecological Therapeutics

**Figure 5:**
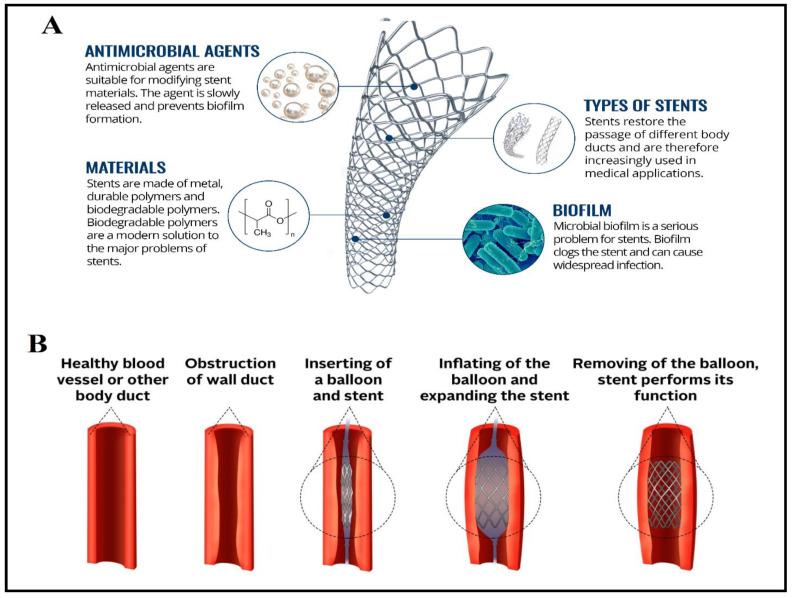
Medical stent: (**A**) properties of biopolymer-based stent, and (**B**) the principle of inserted biopolymeric stent. Adapted from Škrlová et al. [127].

Renal colic is a common condition among pregnant women, affecting both the mother and her fetus. It is the most non-obstetric reason for the hospitalization of pregnant women [126]. The biopolymeric stent has been developed to manage this condition during pregnancy. Can et al. [126] found that urgent stent placement during pregnancy was highly effective, reliable, and safe. The authors also reported that the biopolymeric stent had a low complication rate and was significantly effective in managing the persistent flank pain in pregnant women. Conventional stents have several major issues, such as stent clogging (due to microbial biofilm) and stent migration. Besides removing the stent operatively from the patient’s body, biopolymeric stents have been reported to disintegrate into basic substances, which decompose in the human body, without any need for surgical removal [127]. Simultaneously, biopolymer-based stents have the potential to cross-link with different antimicrobial agents, drugs, or antibiotics to prevent biofilm formation and on their surface (Figure 5) [19]. The antimicrobial agents are released slowly during the degradation of the bio-stent, which prevents the adhering and growth of bacteria and avoids the formation of biofilm and subsequent stent clogging [126].

**Figure 6:**
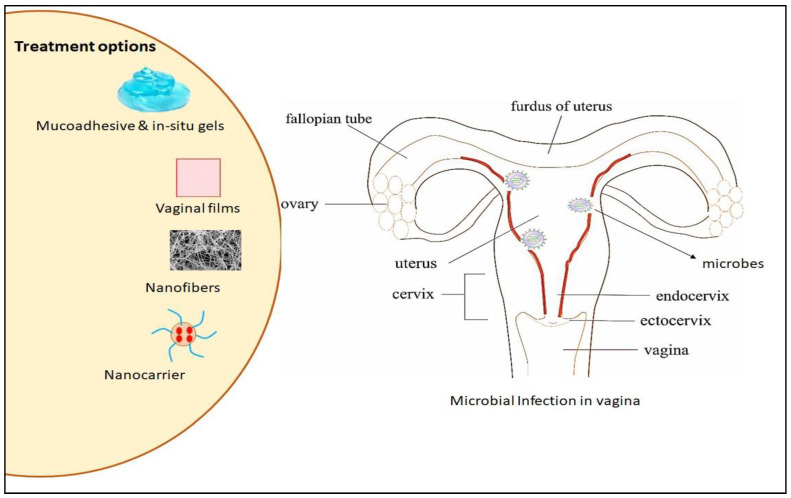
Natural biopolymer-based options in the treatment of vaginal microbial infections. Adapted from Pandey et al. [135].

Several bioadhesive biopolymers have been fabricated in the past few years for different mucosal sites, including the vagina, to treat several gynecological diseases. Cazorla-Luna et al. [128] prepared vaginal mucoadhesive bilayer films using ethylcellulose as a precursor material for controlled release of the antiviral drug antiviral. The prepared film showed sustained antiviral drug release with more than 360 h mucoadhesion time, without any signs of toxicity. The authors revealed that biopolymers offer a promising option for women of self-protection against various sexually transmitted diseases such as HIV. Thanks to normal flora such as lactobacilli, the pH of the vaginal fluid between 4 and 5.5 in healthy women are considered a drug delivery site for numerous drugs [129]. Unlike conventional polymeric vaginal drug delivery systems, which are associated with some drawbacks—including toxicity, potential allergic action, messiness, and leakage—in addition to relatively poor retention time [130]. Biopolymers are valuable candidates for numerous mucoadhesive gel formulations that can be used in drug delivery due to their proven safety [131], high biocompatibility [106], ability to conjugate with a variety of drugs and other polymers [132], and eco-friendliness [107]. Women of reproductive age are increasingly prevalent in terms of vaginal microbial infections. Several novel approaches have been used to fabricate biopolymer-based materials, including nano-systems, vaginal films, mucoadhesive polymeric systems, nanofibers, and smart stimuli-responsive systems [133,134]. Biopolymers-based materials have been used to combat the limitation of conventional synthetic polymeric products, such as low retention time, discomfort, lack of optimal prevention, and treatment approaches that led to a high recurrence rate for vaginal diseases [135]. Figure 6 summary of biopolymer-based therapeutic options for vaginal microbial infection. 

The elastin-like polypeptide has been used to measure the effects of elastin-like polypeptide polymer size on pharmacokinetics in addition to biodistribution and placental transfer of the biopolymer [136]. The authors revealed that pharmacokinetics and biodistribution of an elastin-like polypeptide during pregnancy were size-dependent. The biopolymer was too large to traverse the placental barrier. Verifying that elastin-like polypeptide fusion is a powerful method that can modulate half-life and prevent cargo molecules’ placental transfer [136]. These biopolymers can also deliver certain drugs during pregnancy, preventing the drug from fetal exposure while targeting the pregnant mother.

### 3.3. Biopolymer-Based Materials in Obstetrics and Gynecological Surgical Sutures

**Figure 7:**
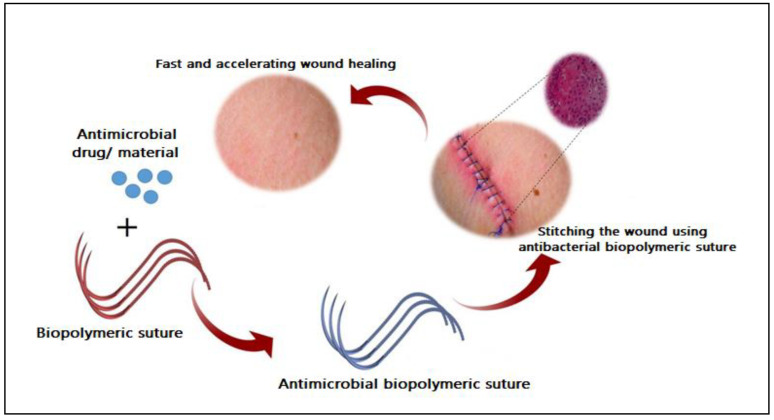
Schematic illustration of developing and using antibacterial biopolymeric based suture in obstetric and gynecological operations. Adapted from Joseph et al. [139].

Throughout the history of sutures and surgery related to obstetric and gynecologic, various materials have been used, including wires of gold, silver, and iron: animal hairs; dried gut; silk; plants fiber such as tree bark; more recently, biopolymer-based materials start attracting great attention for developing different wound closure. However, no study has specified the best and perfect suture material for all situations [137]. Gynecological surgeries have great potential for adjunct vascular interventions, especially in women who suffer from obesity or do not do enough exercise, given the proximity of major and main intra-abdominal and pelvic blood vessels [138]. Levin et al. [138] recently reported that vascular repairs in gynecologic operations have become uncommon due to the great advancement in medical and material science. Still, it predicts major morbidity and mortality as it can dramatically turn to become a critical and life-threatening issue. Biodegradable sutures have received significant attention in antimicrobial delivery and wound healing applications [19]. Surgical sutures have been a reliable and effective strategy for preventing wound infection in post operations. Smart surgical sutures have developed from biopolymers with antimicrobial properties (Figure 7), delivering various antibacterial and anti-inflammatory drugs and natural materials to the surgical site [139]. 

Natural biopolymers’ role in the fabrication of various biocompatible and reliable surgical sutures with good antimicrobial and mechanical properties is paramount, making them appropriate and highly preferred for vascular repairs in gynecological operations [140]. Recent research has focused on developing enhanced sutures that possess improved functionalities, which could play a prominent role in obstetric and gynecological operations [141,142]. The braided corrugated vascular prosthesis has been fabricated from poly-lactic acid and polyethene terephthalate by Fangueiro et al., who patented this technology [106]. The braided corrugated vascular prosthesis showed great advantages in re-establishes blood flow in all affected and damaged segments of blood vessels, which have been used in many vascular surgeries [143].

### 3.4. Biopolymer-Based Materials for Obstetrical and Gynecological Wound Management

**Figure 8:**
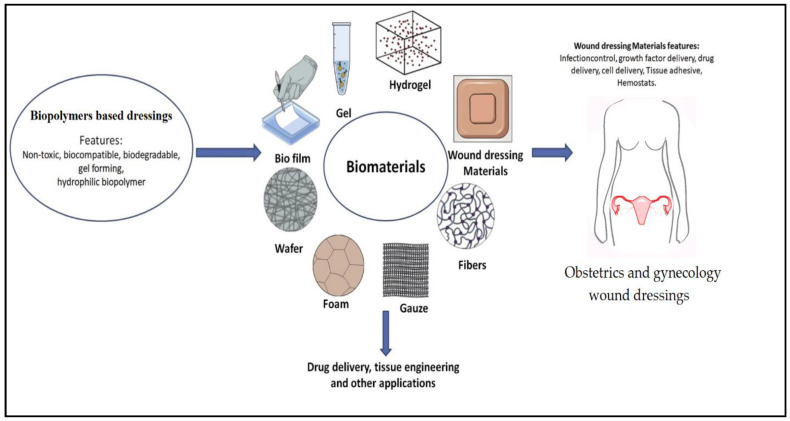
Biopolymeric-based wound dressings for obstetric and gynecological wound infections. Adapted from Varaprasad et al. [156].

Wound healing disorders in obstetrics and gynecology are among the medical professions’ issues due to their chronicity, difficult-to-heal, serious complications, extended hospitalization times, and increased treatment costs [144]. Wounds in obstetrics and gynecology have been classified by the Centers for Disease Control and Prevention (CDC) as clean-contaminated wounds [145]. Plowman et al. [146] reported an overall incidence of healthcare-associated infections of 7.8%, which varied based on the type and specialty. Gynecology was the highest incidence with 13.1%, while the obstetric incident was the third with 10.1%. In a different study, Johnson et al. [147] reported more than 10% of women undergoing natural vaginal delivery and who sustained perineal trauma, which required suturing, developed a wound infection, which could be even increased in women who suffer from obesity, chronic diseases such as diabetes [148,149]. In particular, cesarean section and abdominal hysterectomy, surgical site infections rates are 1.8–11.3% and 3.0–12.2%, respectively, reported to be much higher 21–39% in women who undergo surgical treatment of tumors of the vulva [150,151]. Stanirowski et al. [152] reviewed the available literature. They discussed the possibilities for using efficacy and low-cost growth factors in treating post-surgical wounds in obstetrics and gynecology and revealed significant difficulties in healing these wounds even with growth factors and hormones. The prevention of infections in obstetrics and gynecology is challenging. The vagina and cervix’s normal flora can promote serious infection under certain circumstances in females’ genital tract [153]. In the past few years, the results of numerous clinical trials regarding using biopolymer-based wound dressing on different wounds of obstetrics and gynecology attracted more scientists for use of biopolymers in wound dressing. Fouda et al. [154] treated cotton with two biopolymers namely chitosan and linear polyvinyl amine, which possess antimicrobial activity for sensitive wound treatment applications such as diabetic, obstetrics, and gynecology wounds and revealed synergistic bacteriostatic effect for the treated cotton. Kamoun et al. [155] reviewed the potential use of biopolymeric hydrogel membranes to dress different types of wounds. They reported that these biopolymers could fulfill the demanded conditions required for dressing and treatment of skin wounds. Figure 8 presents the types of biopolymeric dressings and their advantages in dressing obstetric and gynecological wounds.

Natural delivery has also been associated with vaginal trauma caused by vaginal surgery, leading to serious wound infection [156]. Various cellulose and chitosan-based biocomposites have been developed and characterized to explore their potential use in obstetric and gynecological tampons [157]. Viscose fibers have been coated by chitosan as an antibacterial agent to develop gynecological tampons, which dissolve in acetic or lactic acids to inhibit microbial growth and adjust its pH [158]. This biopolymer-based tampon proved to be better than many commercial ones. The high absorption rate and the antimicrobial properties of chitosan make it highly suitable and beneficial for pregnant women [157]. As natural polymers, chitosan’s ability to cross-link with cellulose fibers in regular biodegradable textile sheets allows the fabrication of smart wound dressings highly suitable for adsorption, preventing microbial growth, and accelerate wound healing.

## 4. Issues and Challenges of Biopolymers in Obstetrics and Gynecology

Even with promising trends of biopolymers for applicability, they still need to be improved to suit the desirable properties for obstetrics and gynecology. Few disadvantages have been reported upon using some biopolymers, which differed based on the type of application and the type of biopolymers. Many biopolymers possess rapid degradation rates and low mechanical properties [159], which may not be desired in some gynecological applications such as early pregnancy stents. Some researchers have proposed using hybrids of biopolymers to overcome this issue, and they were able to enhance the mechanical properties significantly and delay the rate of degradability. High hydrophilic capacity is another issue reported in some literature [106], which may not be desirable for the humid environment such as the vagina or in vivo applications. The properties of different biopolymers can be significantly enhanced to meet the needs for any desired application. Still, the cost of production may not be effective and reliable for commercialization purposes. It is necessary to conduct more enhancements and economic studies, enhance the currently developed materials, develop new ones, overcome the cost-effective challenge, contribute to scientific knowledge, and, consequently, contribute to future generations and ensure sustainability. A major challenge associated with designing new biopolymer-based mucoadhesive is studying the interactions between the new formulation and mucosal fluids/tissues, monitoring the long-term effect, and bio distribution of the formulation content upon vaginal administration. 

## 5. Conclusions

The overuse of conventional polymer-based materials led to serious health issues for women and babies in the current and future generations due to their ability to induce genetic alteration, in addition to the generation of a large volume of non-degradable wastes. The technological advancements in materials science, engineering, and medical professionals have stimulated the search for safer and better alternatives to sustainability goals. The safer alternatives should be non-hazardous to women, their babies, and the environment. Biopolymers have been used in numerous medical applications, including obstetrics and gynecology. They deal with sensitive parts on the women’s body and fetus, as safer alternatives for synthetic polymers, due to their extraordinary and unique properties. Many biopolymer-based diagnostic and therapeutic materials have been developed and proven safe for pregnant women and future babies. Obstetric and gynecologic specialists or surgeons who use screening, examination, and operation materials, should benefit from a better understanding of the properties, health, and environmental effect of each type, the underlying principles of biopolymer-based materials for different obstetric and gynecological applications may discover various advantages and benefits to the use of such materials.

## Figures and Tables

**Table 1:** Advantages and disadvantages of biopolymer-based formulations in cosmetic and personal care.

Biopolymer	Advantages	Disadvantages	Ref
Cellulose	Improve the moisture in the skin and minimizes hyper-pigmentation appearance.	Poor compatibility with hydrophobic matrixes.	[120]
Chitosan	Strong antimicrobial, antioxidant properties, as well as softens the skin.	Chitosan intrinsic properties may be affected by its cross-linking.	[121]
Gelatin	Improving skin health and significantly cause skin firmness.	Potential allergic reactions in some individuals.	[122]
Hyaluronic acid	Reduction of wrinkles and visibility of fine lines, as well as smoothening the skin.	Rash on the application site and potential allergic reactions.	[123]
Collagen	Reduces skin wrinkles, improves its elasticity, and boosts skin hydration.	Possible inflammation responses in some individuals.	[124]
Alginate	Improve skin elasticity, strengthens and freshens, as well as erasing fine wrinkles.	Some formulations may have a foul smell.	[125]

## Data Availability

This study did not report any data.
